# NO donors and NO delivery methods for controlling biofilms in chronic lung infections

**DOI:** 10.1007/s00253-021-11274-2

**Published:** 2021-05-03

**Authors:** Yu-Ming Cai, Ying-Dan Zhang, Liang Yang

**Affiliations:** 1 Institute for Life Sciences, University of Southampton, Southampton, SO17 1BJ UK; 2 School of Medicine, Southern University of Science and Technology, Shenzhen, 518000 China

**Keywords:** Nitric oxide, Biofilm, Chronic lung infection, *Pseudomonas aeruginosa*

## Abstract

**Abstract:**

Nitric oxide (NO), the highly reactive radical gas, provides an attractive strategy in the control of microbial infections. NO not only exhibits bactericidal effect at high concentrations but also prevents bacterial attachment and disperses biofilms at low, nontoxic concentrations, rendering bacteria less tolerant to antibiotic treatment. The endogenously generated NO by airway epithelium in healthy populations significantly contributes to the eradication of invading pathogens. However, this pathway is often compromised in patients suffering from chronic lung infections where biofilms dominate. Thus, exogenous supplementation of NO is suggested to improve the therapeutic outcomes of these infectious diseases. Compared to previous reviews focusing on the mechanism of NO-mediated biofilm inhibition, this review explores the applications of NO for inhibiting biofilms in chronic lung infections. It discusses how abnormal levels of NO in the airways contribute to chronic infections in cystic fibrosis (CF), chronic obstructive pulmonary disease (COPD), and primary ciliary dyskinesia (PCD) patients and why exogenous NO can be a promising antibiofilm strategy in clinical settings, as well as current and potential *in vivo* NO delivery methods.

****Key points**:**

• *The relationship between abnormal NO levels and biofilm development in lungs*

• *The antibiofilm property of NO and current applications in lungs*

• *Potential NO delivery methods and research directions in the future*

## **Introduction**

The past century witnessed the successful fight against many acute bacterial infections in lungs with the discovery of different antibiotics, such as life-threatening pneumonia which can now be cured with proper antimicrobial therapy. However, slower-progressing chronic lung infections are now affecting the life quality of millions of people (Bjarnsholt 2013; Quaderi and Hurst 2018; Kelly 2017). These infections are often associated with bacterial biofilms, a growth mode where multiple bacteria stick together and form a consortium. Compared to the free-floating planktonic state, this growth mode provides encased individual cells much higher tolerance to antibiotics, leading to chronic symptoms and therapy failures. With the slow progress in finding new antibiotics, antibiofilm agents can serve as an adjunctive therapy in chronic lung infections, enhancing the susceptibility of bacterial cells towards current antibiotics and increasing life expectancy (Li and Lee 2017).

One antibiofilm agent that attracted much attention is nitric oxide (NO). NO is endogenously produced by many types of cells in the airway and regulates different cell behaviours. Particularly, NO plays a crucial part in innate immunity against a small inoculum of inhaled bacteria, where a high concentration (above micromolar) of NO generates reactive nitrogen oxide species (RNOS) by the reaction with oxygen or superoxide. RNOS exert bactericidal effects *via* damaging bacterial DNA, inhibiting enzyme functions and inducing lipid peroxidation (Schairer et al. 2012; Darling and Evans 2003; Sivaloganathan and Brynildsen 2020). In the past 15 years, NO was also found to inhibit bacterial biofilms in different settings at much lower, nontoxic concentrations (pico–nanomolar) (Barraud et al. 2015). As such, the dual functions of NO can both be used to fight against lung infections. However, in CF, COPD and PCD patients, pathogens cannot be cleared out by abnormal levels of endogenous NO, resulting in chronic infections. Exogenous NO successfully reduced biofilms and potentiated the efficacy of conventional antibiotics in CF patients (Howlin et al. 2017), suggesting it may also be a promising antibiofilm agent in other lung diseases. Here, we summarize current knowledge of the abnormal NO levels in CF, PCD and COPD patients, the efficacy of NO on biofilms formed by chronic lung infection-related pathogens, and the existing devices for exogenous NO applications. The aim is to trigger more investigations into the compromised NO pathways in different patients and improve the efficiency of NO application in chronically infected airways.

## Chronic infections in different pulmonary diseases

Chronic lung infections can develop in patients suffering from certain diseases or conditions that cause deficiency in their innate immunity. For example, a compromised first line barrier of lung epithelium cells are often found in chronically infected lungs. The epithelium of bronchial wall is covered by cilia, on top of which residents the mucus (Boyton and Openshaw 2002). The outer layer of mucus is a viscous gel phase comprising a mixture of water, glycoproteins, immunoglobulins, lipids, and electrolytes, whilst the inner layer, directly in contact with cilia, is a fluid or sol phase (López and Martinson 2017). Mucus is continuously swept back up through the lungs by the movement of cilia (ciliary beating), referred to as mucociliary transport (López and Martinson 2017). This mechanism allows inhaled bacteria that are trapped in the mucus layer to be cleared out and ensures that pathogens do not directly contact with the epithelial cells or reach the alveolar cavities (Fliegauf et al. 2013), However, a deficient mucociliary transport is found in cystic fibrosis (CF), chronic obstructive pulmonary disease (COPD) and primary ciliary dyskinesia (PCD) patients. PCD is an autosomal recessive genetic disorder causing defects in ciliary biogenesis, structure, and function (Wijers et al. 2017; Horani et al. 2016). Such malfunctioning cilia are unable to move properly and propel mucus, leading to a progressive accumulation of mucus and the failure of mucociliary transport. In contrast, CF is caused by mutations in the cystic fibrosis transmembrane conductance regulator (CFTR) gene, which is translated into proteins function as chloride channels in surface airway epithelial cells and the cells of the submucosal glands. The lack/dysfunction of this channel leads to insufficient chloride transport followed by reduced water secretion. The failure to maintain proper salt–water balance results in abnormal periciliary fluid depth and the production of highly viscous mucus that remain tethered to gland ducts, interfering with ciliary movement and contributing to the failure in mucociliary transport (Chmiel and Davis 2003; Hoegger et al. 2014). Smoking and exposure to noxious gases and particles are the leading causes of COPD, which can reduce both the number and length of cilia, enlarge the submucosal glands, and increase the number of goblet cells, leading to mucus hypersecretion that further interfere with ciliary motion (Simet et al. 2010; Rogers 2005).

Despite the different aetiologies, chronic bacterial infection is the main reason for decreased life quality and shortened life expectancy of these patients. Some bacteria involved in these infections can survive treatments that are predicted to eradicate them, where conventional antibiotics lose their efficacies. These infections may persist lifelong in some patients. The therapy failure leads to a vicious circle—damaged lungs fail to resist new infections, whilst new infections further damage lung tissue. The most prevalently found pathogens in CF lungs range from *Haemophilus influenzae* and *Staphylococcus aureus* as early colonisers in children, to *Pseudomonas aeruginosa* and *Burkholderia cepacian-complex* frequently isolated from adults (Surette 2014). *H. influenza*, *Streptococcus pneumonia*, *P. aeruginosa*, *Moraxella catarrhalis*, *Haemophilus parainfluenzae* and *S. aureus* are frequently found in stable COPD patients (Beasley et al. 2012). For PCD patients, nontypeable *H. influenzae* (NTHi) is the most commonly isolated species, followed by *P. aeruginosa*, *S. aureus*, *S. pneumoniae*, and *M. catarrhalis* (Alanin et al. 2015). Why can’t these patients clear the infection?

## Biofilms in chronically infected lungs

### *P. aeruginosa* aggregates in CF respiratory tracts

**Fig. 1:**
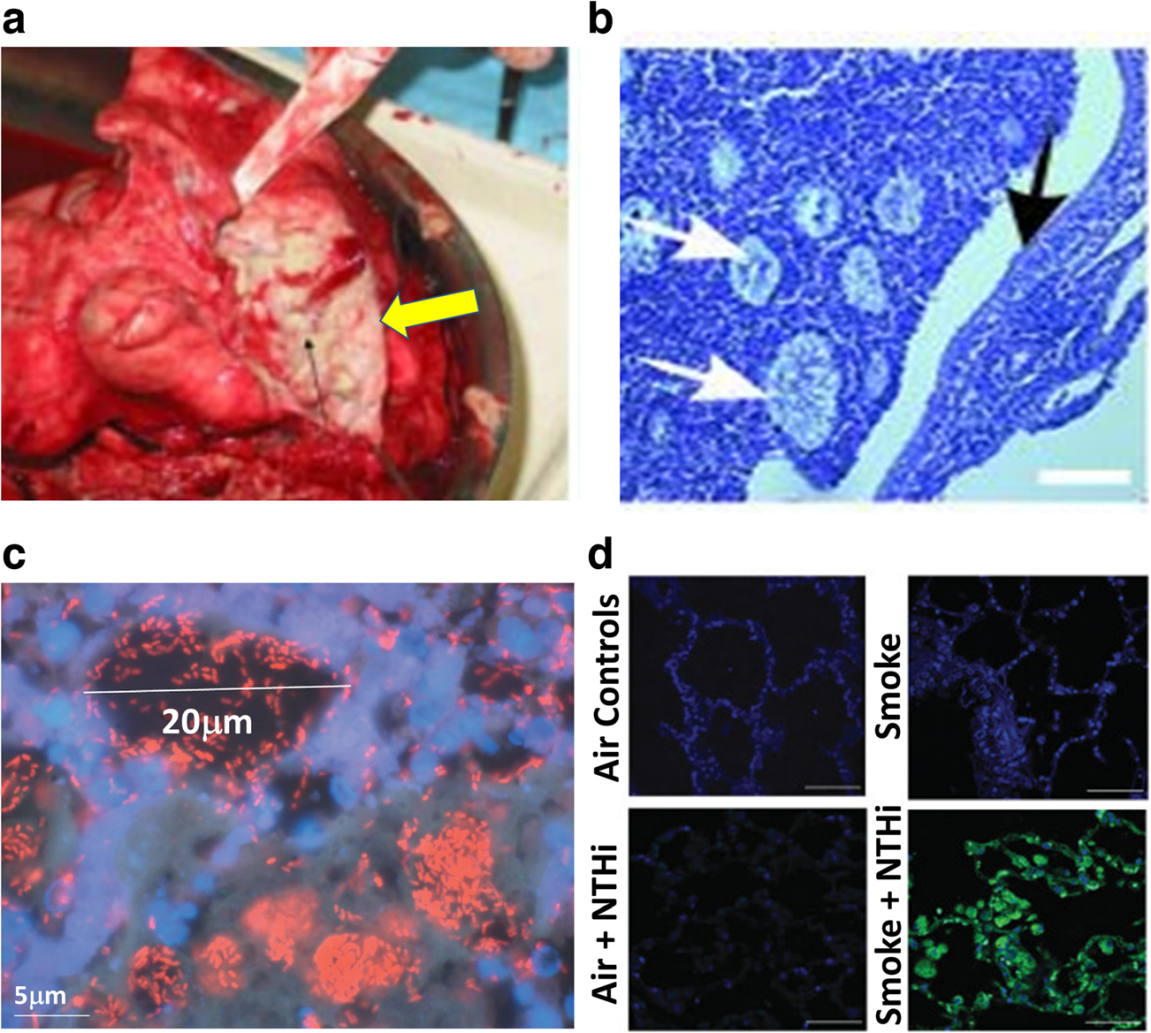
**a** Green mucus (yellow arrow) is filling the major airways of a newly explanted CF lung (Bjarnsholt et al. 2009). **b** Thin section of an obstructed CF bronchus. Aggregated biofilms are not attached to epithelial surface (black arrow) but are embedded within intraluminal material (white arrows). Scale bar=100μm (Worlitzsch et al. 2002). **c** Intraluminal *P. aeruginosa* aggregates surrounded by PMNs stained with PNA FISH and DAPI (Bjarnsholt et al. 2009). **d** NTHi forms massive aggregates within the lungs of a COPD ferret after infection and smoke exposure, whilst the lungs exposed to air display only few punctate NTHi. Nuclei were stained with DAPI (blue), and bacteria were stained with anti-NTHi polyclonal antibodies conjugated to Alexa 488 (green). Scale bar=50μm (Hunt et al. 2020). Permissions to reuse images were obtained from journals and/or Copyright Clearance Centre.

Aggregated biofilms are now recognised as the cause of many chronic infections. Specific to chronic lung diseases, the initial observation and the very first micrograph of a slimy biofilm in CF sputum was published in 1977 (Høiby 2017, 1977). Many later clinical examinations repeatedly confirmed the existence of biofilms/aggregates in CF sputa and the lumen of CF lungs (Worlitzsch et al. 2002; Bjarnsholt et al. 2009; Bjarnsholt et al. 2013) (Fig. 1). Compared to single celled bacteria freely floating in aqueous environments, which are more formally named as “planktonic cells”, aggregates or biofilms are bacteria physically stuck together and encased in the self-produced polymeric matrix containing proteins, DNA and polysaccharides (Flemming and Wingender 2010). The protective shield and united lifestyle grant the embedded single cells much higher tolerance to innate host defences (Leid 2009). Neutrophils that settle onto aggregates are unable to migrate away from the point of contact, and aggregates in mucus are frequently founded to be surrounded by dead or dying neutrophils. They are unable to penetrate but are instead killed by the pathogens (Downey et al. 2009). Consequently, biofilms that escaped the attack from innate immunity are persistently established *in vivo*. With the prevalence of CF (more than 70,000 worldwide with ~1000 new cases diagnosed per year) and the dominant role of *P. aeruginosa* in the later stage (>80% of CF adults are entangled with *P. aeruginosa* chronic infection), *P. aeruginosa* biofilm in CF patients inevitably became the most extensively chronic lung infection model.

**Fig. 2:**
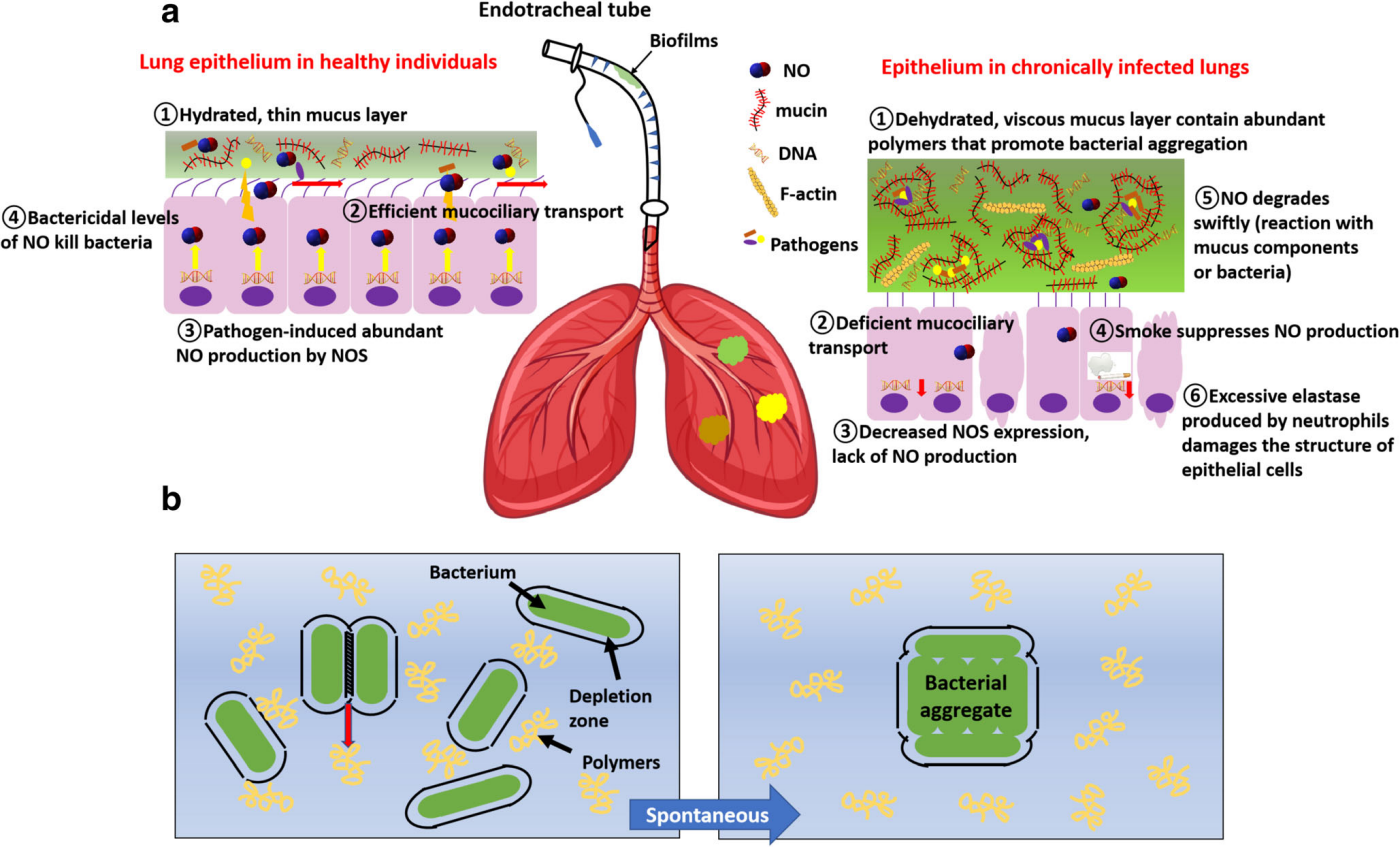
**a** Schematic diagram of biofilms/aggregates in chronically infected lungs and possible mechanisms of how abnormal levels of NO are associated with chronic lung infections. **b** Schematic diagram of depletion aggregation. Bacteria (green spherocylinders); polymers (yellow twisted lines); and depletion zones (dashed lines around the bacteria). When two bacterial cells come closer, polymers in between are squeezed into the solution (red arrow). Such spontaneous aggregation minimizes the depletion zones occupied by bacteria and maximizes the entropy of the system (figure adapted from Dorken et al. 2012). The icons of lung, DNA, and cigarette were obtained from vectors available from Vecteezy.com

Invading, planktonic *P. aeruginosa* cells first adhere reversibly to the gel phase of the viscous mucus layer in CF patients using flagella and pili, followed by cell division and the formation of small clusters as early biofilms/aggregates (Høiby 2002). The dehydrated, concentrated mucus plays a crucial role in the formation of macrocolonies in CF airways—an *in vitro* comparison between normal (2.5% solid) and CF-like (8% solid) mucus showed that with the same initial inoculum and end point CFU, biofilms were only detected in CF-like mucus (Matsui et al. 2006) (Fig. 2a). Aggregates formed in the thick mucus layer may be promoted by a mechanism called “depletion aggregation” (Secor et al. 2018), which was postulated to be mediated by entropic forces between uncharged/like-charged polymers and bacteria. The thick CF mucus layer contains abundant polymers such as mucin, DNA, and F-actin (all negatively charged), as well as bacteria particles. In such a mixture, polymers cannot reach the surface of bacteria by a distance less than the size of its own radius. Each bacterial cell is therefore surrounded by a “depletion zone”. When two bacteria cells come closer, the depletion zones overlap, constraining the presence of polymers in between. The polymer concentration between cells is therefore essentially 0, whilst the osmotic pressure generated by different concentrations of polymers across the cells can physically hold cells together. This spontaneous aggregation reduces the total depletion zones and produces more space for polymer movement, hence maximizing the entropy of the system following the second law of thermodynamics (Fig. 2b). As such, it is hypothesized that in the presence of abundant polymers, bacteria can quickly aggregate through this passive physical method without the positive involvement of bacterial activities (Dorken et al. 2012; Secor et al. 2018). At this initial stage, *P. aeruginosa* still continues its nonmucoid phenotype (Chmiel and Davis 2003). However, *P. aeruginosa* that survived the phagocytosis may start benefiting from its end products. For instance, oxygen radicals produced by polymorphonuclear leucocytes (PMNs) as a result of an inflammatory response induces mutations in surviving *P. aeruginosa*, such as in gene *mucA* (Høiby 2002). Such adaptation leads to excessive alginate production, and this adhesive substance can promote bacterial aggregation, offering stronger protection against both host immune defence and antibiotic delivery. On the other hand, the remaining bacteria stimulate repeated and excessive inflammatory responses, especially the progressive accumulation of neutrophils, which in turn results in overproduction of elastase (Laval et al. 2016). An excessive amount of elastase can degrade a range of cell surface receptors on lymphocytes, neutrophils and dendritic cells that are important for pathogen recognition and digestion (Gifford and Chalmers 2014) and also destructs lung cell tissues causing structural damage that further cripples the clearance process (Chmiel and Davis 2003). Altogether, the thick mucus layer, the *in vivo* bacterial pathoadaptation, and tissue damage all contribute to the long-term bacterial residence and the development of aggregated biofilms.

Another hallmark of biofilms is the high tolerance to antibiotics provided by this lifestyle besides the intrinsic or acquired resistance, resulting in the chronic pathology in CF patients despite the routine applications of antibiotics. Traditionally, the terms “tolerance” and “resistance” were used interchangeably in many biofilm-related articles. Here in this review, these two terms are distinguished based on their different mechanisms. Resistance is due to intrinsic or acquired genotypes that allows bacteria to survive antibiotic treatments even at planktonic states, which is usually characterised as increased minimum inhibitory concentration values. In contrast, tolerance in biofilms is due to phenotypic changes offered by the formation of biofilms, where cells survive high concentrations of antibiotics only if embedded in biofilms. If cells in biofilms are reverted to planktonic state, they are sensitive to antibiotics (For more details please see review articles from Stewart 2015 and Ciofu and Tolker-Nielsen 2019). Apart from the different responses to antibiotics offered by growth modes, how biofilms are grown may also significantly affect the efficacy of antibiotics. Clinical examinations observed that bacterial aggregates found *in vivo* are generally much smaller in size compared to surface-attached biofilms grown in laboratory, suggesting a different physiological character under the pressure of host response, ROS, and nutrient limitation (Bjarnsholt 2013). For example, aggregates embedded in the mucus plug are surrounded by PMN cells which consume a large proportion of oxygen, resulting in a lack of access to oxygen for *P. aeruginosa* cells (Kolpen et al. 2014). Aggregates are therefore grown in anaerobic conditions, leading to different metabolism and antibiotic tolerance mechanisms compared to biofilms grown in classic flow-cell systems or microtiter plates. As such, *in vitro* determination of antibiotic dosage may not accurately reflect the requirement for biofilm elimination in patients. The repeated usage of some antibiotics in patients that are not sufficient to eradicate biofilms may present a selective stress that favour the mutations related to antibiotic resistance, and even the component of CF sputum can cause an accumulation of mutations in the *lasR* gene that increase the tolerance to β-lactam antibiotics (Marvig et al. 2013; Clark et al. 2018; Azimi et al. 2020). Hence, both physiological tolerance and hereditary genetic mutations offered by the biofilm mode of growth make it almost impossible to eradicate bacterial aggregates settled down in the mucus layer, even with aggressive combinational antibiotic treatments (Soren et al. 2019).

### Biofilms/aggregates in the respiratory tract of PCD and COPD patients need further investigation

Compared to extensively studied chronic infections in CF, biofilms in PCD and COPD patients with chronic bacterial infection and massive mucus production appeared to attract much less attention, despite a wealth of publications generally claimed that the formation of biofilms contribute greatly to the difficulties in the treatment of chronic lung infections. For instance, it was reported that bacterial evolution in PCD and CF patients follows a similar pattern and the biofilm formation ability of *P. aeruginosa* clinical isolates from PCD sputa was tested *in vitro* (Sommer et al. 2016), but no micrograph was available to confirm the aggregates in sputum samples. *H. influenzae* is the most frequent microorganism isolated from a range of PCD patients (Alanin et al. 2015), yet it was only confirmed *in vitro* that *H. influenzae* cells are more likely to form aggregates on the PCD epithelial cells than healthy control (Walker et al. 2017). Biofilm growth mode has long been implicated in COPD despite the lack of direct evidence, and this hypothesis was only very recently supported by the detection of *H. influenzae* multicellular aggregates within the airway lumen of COPD ferret (Hunt et al. 2020). Models of chronic lung infection including the implication of microbiota and biofilms have been set up, yet many more investigations are required to consolidate the biofilm growth mode in respiratory tracts of different patients. Assuming that multicellular biofilms can be prevalently found in all these patients, many questions still await to be answered for better treatment strategies. For example, the dominant species found in different patients varies; are all aggregates residing in mucus layers or some are attached to the epithelium? Is there any difference in the size and amount of aggregates observed in different type of patients, and are they correlated to the severity of diseases? How do these *in vivo*/*ex vivo* aggregates respond to antibiotics or other treatments?

### Biofilm-related infections during mechanical ventilation

Another special type of biofilms in lung infection occurs during invasive mechanical ventilation, which has been frequently used in very severe acute exacerbation COPD patients. Despite being a life-saving procedure for critically ill patients, intubation with endotracheal tubes (ET tubes) provides perfect landing sites for bacterial adhesion and biofilm formation on both the inner luminal and outer surface (Li et al. 2015; Cairns et al. 2011). The colonisation of microorganisms and the formation of biofilms on ET tubes can be rapid, within hours after insertion (Vandecandelaere et al. 2012), and by the end of 10 days almost all the ET tubes are covered by biofilms. A close link was observed between the microbial flora of ET tube biofilms and the microorganisms involved in the onset of ventilator-associated pneumonia (VAP), including frequently identified multidrug-resistant ESKAPE pathogens (Vandecandelaere and Coenye 2015). Biofilms on ET tubes are therefore heavily responsible for VAPs, resulting in higher mortality rates and much longer ICU- and hospital-lengths of stay (Diaconu et al. 2018). More importantly, biofilms on ET tubes also show great tolerance under the challenge of antibiotics like those grown on plastic plates *in vitro*, requiring novel combinational therapeutic strategies for an efficient biofilm treatment in VAP patients (Gordon Sahuquillo et al. 2015).

## Abnormal levels of NO are associated with chronic lung infections

Apart from the mucociliary transport, lung epithelium cells are also primary sources of NO (Lane et al. 2004). Endogenously produced NO in the airways is evidenced by its appearance in the exhaled breath. NO is synthesized from l-arginine by NO synthase (NOS) with three isoforms—endothelial NOS (eNOS), neuronal NOS (nNOS), and inducible NOS (iNOS). Both eNOS and nNOS are constitutively expressed and can generate picomolar levels of NO in pulmonary endothelial cells and nonadrenergic, noncholinergic inhibitory neurons, respectively (Xu et al. 2006). The iNOS is continuously expressed under basal airway conditions, and can be induced by inflammatory stimuli during bacterial infection to produce bactericidal levels of NO (Xu et al. 2006; Guo et al. 1995). The total production of NO from these NOSs can be reflected by fractional exhaled nitric oxide (FENO) quantifying the level of nitric oxide gas in the breath (Miskoff et al. 2019; Duong-Quy 2019).

However, much lower FENO levels are frequently found in CF patients (Elphick et al. 2001; Korten et al. 2018; Grasemann et al. 1997; Keen et al. 2010; Thomas et al. 2000). Bronchial epithelium from CF lungs showed less immunohistochemistry stain for iNOS compared to controls, and further *in situ* hybridization also showed less iNOS mRNA in CF epithelium than in controls (Meng et al. 1998). When a differentiated human bronchial epithelial cell line derived from CF patient is transfected with human iNOS cDNA, reduced *P. aeruginosa* adherence and enhanced killing of internalised bacterial cells were observed (Darling and Evans 2003). Also, genetic variations in the baseline nos1 (nNOS) lead to an altered NO production (Texereau et al. 2004; Grasemann et al. 2002; Grasemann et al. 2000), and CF patients harbouring nos1 genotypes are much more susceptible to *P. aeruginosa* colonisation (Grasemann et al. 2002). These all suggest that in CF patients, the low level of NO may play an important role in the higher susceptibility towards pathogens.

Epithelium is not the only NO resource during an inflammation in the airway, and different immune cells can also produce NO in response to pathogenic stimuli. It was found that PMN cells in freshly expectorated sputum samples from CF patients with chronic *P. aeruginosa* infection can actively produce NO (Kolpen et al. 2014). However, NO is an unstable free radical, and once released into the aqueous or gel phase of the thick mucus layer comprising a vast number of biological components, it might degrade quickly before reaching the bacterial cells. This postulation was supported by some findings where high concentrations of the stable NO metabolites nitrate and nitrite were detected in CF sputum, despite the lowered FENO (Grasemann et al. 1998; Jones et al. 2000). These stable end products of NO accumulated in mucus may in return favour the growth of *P. aeruginosa* in anoxic environment through denitrification process, promoting the chronic infection (Zumft 1997).

A marked reduction in nasal NO (nNO) and FENO was repeatedly found in PCD patients compared to healthy controls (Walker et al. 2012; Walker et al. 2013; Lucas and Walker 2013; Shapiro et al. 2017; Zhang et al. 2019a; Horváth et al. 2003). Despite NTHi being the leading pathogen in PCD, several hypothesis were raised to explain the reduced NO in PCD similar to in CF, including increased breakdown of NO trapped in paranasal sinuses, and lowered expression/function loss of NOS. However, with conflicting data from different publications, the underlying mechanisms still remain unclear (Walker et al. 2012). An early study showed that NO metabolites were not decreased in PCD exhaled breath condensate, suggesting the existence of NO in the secretion (Csoma et al. 2003). A later study compared the expression of NOS amongst children with PCD and with secondary ciliary dyskinesia (SCD, comparably infected and inflamed control group who did not have a congenital defect of ciliary function) (Pifferi et al. 2011). The expression of iNOS was significantly lower in children with PCD compared SCD group, and a positive relationship between iNOS gene expression and nNO levels was demonstrated, implying an impaired expression of iNOS may count for lowered NO output in PCD patients. A lack of substrate l-arginine may also contribute to the lack of NO production, as administration of l-arginine increased nasal and exhaled nitric oxide levels in PCD patients (Grasemann et al. 1999; Loukides et al. 1998). Despite the lack of direct evidence, such lowered NO in PCD patients may further compromise the nonspecific defences against pathogens in the airway on top of deficient ciliary movement, contributing to recurrent sinopulmonary infection. A particularly interesting study was conducted by a healthy researcher with no history of sinus problem, where he developed sinus infection after the nasal application of NOS inhibitors in his right nostril with a marked reduction of NO, whilst the untreated left nostril remained unaffected (Lundberg 2005). Although this experiment was conducted in nasal, the results may support the general notion that NO plays an important role in primary host defence in the upper airways.

The relationship between NO and COPD is much more complicated with the involvement of cigarette smoke, which may interfere with NO pathway in addition to the inflammation process. As COPD patients may have different history of smoking, accordingly, reported exhaled NO levels in COPD were conflictual depending on the subjects and measurement approaches (Corradi et al. 1999; Ansarin et al. 2001; Brindicci et al. 2005; Clini et al. 1998; Rutgers et al. 1999; Maziak et al. 1998). A recent meta-analysis based on a wealth of literature concluded that FENO levels in COPD patients are higher than healthy controls (Lu et al. 2018). This can be supported by previous studies showing that the expression levels of iNOS and nNOS were higher in COPD patients than nonsmokers and smokers with normal lung function (Jiang et al. 2015; Maestrelli et al. 2003; Brindicci et al. 2010). Apart from comparing COPD and healthy individuals, one consensus appears that current smokers exhale less NO than ex-smokers (Corradi et al. 1999; Clini et al. 1998; Malerba et al. 2014; Hynes et al. 2015; Högman et al. 2019). The contribution of cigarette smoke to a lowered FENO in current smoker was postulated to be a downregulation of NOS, or that the oxidants in smoke may damage bronchial epithelial cells and suppress the production of NO (Malerba et al. 2014), or an increased NO consumption in smokers’ airways (Malinovschi et al. 2006). Solid evidence for the effect of smoke on the function of NOS and NO production *in vivo* are yet to be achieved. Interestingly, cigarette smoke extract was shown to inhibit the NOS activity in lung epithelial cells and pulmonary artery endothelial cells *in vitro* (Su et al. 1998; Hoyt et al. 2003), but when mice were exposed to cigarette smoke for either a short time (2–24 h) or 8 months, increased iNOS was found (Wright et al. 2002; Seimetz et al. 2011). Thus, much needs to be investigated for a clearer picture of NO pathway, NO production and consumption, as well as the relationship between NO and the pathogenesis in COPD patients.

Due to the controversial NO output and NOS expression levels in different type of patients, it is impossible to conclude at this stage that chronic infection can be primarily attributed to a deficiency in NO production. However, suffice to say, at least the amounts of functional NO in the respiratory tracts of all these patients are not enough to eliminate the invading pathogens, which subsequently allows for their long-term residence.

## Nitric oxide as an antibiofilm agent

### Low-dose NO disperses biofilms; high-dose NO eradicates biofilms

It was well-documented that NO plays dual functions in humans—at low concentrations, it serves as an intracellular signal regulating a range of cell behaviours; at high concentrations, it exhibits cytotoxicity against tumours and can promote apoptosis. Similar dose-dependent scenarios also apply to bacteria—besides the bactericidal effect at above micromolar scales, NO can prevent the formation of biofilms or disperse established ones as a signalling molecule when administrated at low concentrations (pico–nanomolar). Dispersal is the natural, final stage of biofilm life cycle, where cells are released from mature biofilms for reestablishment at a more favourable niche (Kaplan 2010). Under clinical settings, exogenously added, low concentrations of NO can also trigger this procedure—they reduce the CFU and total biomass of biofilms remaining on the surface by reverting sessile biofilm cells back to free-swimming planktonic state, without posing a lethal effect to bacterial cells within the biofilms. Such action increases the susceptibility of cells encased in biofilm towards other treatments, thus enhancing therapeutic outcomes. For example, by using a traditional NO donor, sodium nitroprusside (SNP), Barraud et al*.* showed that nontoxic dose of NO (500 nanomolar) can trigger the dispersal of mature *P. aeruginosa* biofilms and prevent initial attachment (Barraud et al. 2006). Further studies elucidated that NO modulates *P. aeruginosa* biofilm through regulating a secondary messenger, bis-(3-5)-cyclic dimeric GMP (c-di-GMP), that can be found in most bacterial species (Barraud et al. 2009a; Römling et al. 2013). c-di-GMP is synthesised by a group of enzymes named diguanylate cyclase (DGC), and is hydrolysed by another group of enzymes named phosphodiesterase (PDE). High concentrations of intracellular c-di-GMP promote the switch from planktonic growth modes to biofilm formation (Römling et al. 2013). NO decreases intracellular c-di-GMP levels in *P. aeruginosa via* directly or indirectly stimulating several PDEs, such as NbdA, DipA and RbdA (Barraud et al. 2009a; Morgan et al. 2006; Petrova and Sauer 2012a; Petrova and Sauer 2012b; Li et al. 2013) (See reviews by Cutruzzolà and Frankenberg-Dinkel 2016; Williams and Boon 2019 for detailed molecular mechanisms). Low-dose NO was also proven to prevent or disperse biofilms formed by many different species, although different concentrations and donors were required (Arora et al. 2015; Thompson et al. 2019; Islam et al. 2020). Whilst NO-c-di-GMP pathway was found in *Legionella pneumophila*, *Shewanella oneidensis*, and *Shewanella woodyi* (Carlson et al. 2010; Plate and Marletta 2012; Liu et al. 2012; Fischer et al. 2019), some Gram-positive strains such as *S. aureus* do not contain c-di-GMP related protein domains but can still disperse upon NO challenge, suggesting an alternative signalling cascade is involved and yet to be determined. In contrast, NO released at higher concentrations (micro–millimolar) can eradicate *P. aeruginosa* biofilms by both disrupting the physical structures of biofilms and exhibiting toxicity towards interior cells. NO was shown to decrease the viscoelastic moduli and weaken the mechanical properties of biofilms matrix, possibly through polysaccharide depolymerization and DNA structural damage (Reighard et al. 2015; Rouillard et al. 2020a; Yang et al. 2018; Duan and Kasper 2011; Burney et al. 1999). Structural disruption in biofilms increases the exposure of individual cells towards bactericidal levels of NO, leading to eradication. As such, both low and high dosage of NO treatment can reduce biofilms *via* different mechanisms. Despite the lack of solid evidence of *in vivo* biofilms formed by other leading pathogens frequently isolated from chronic lung infection patients besides *P. aeruginosa*, *in vitro* biofilms formed by these species showed different responses to NO treatment (Table 1). Whilst NO was confirmed to reduce biofilms formed by *P. aeruginosa*, *S. aureus*, *B. cepacian*, and *M. catarrhalis*, NTHi and *S. pneumoniae* appeared unaffected at concentrations that are effective against other species. Whether this resistance comes from some intrinsic mechanisms of these two species, or it is more of the nonoptimised experimental procedures or NO resources remains unknown. Deeper studies into the effect of NO on different species, especially clinical isolates, with more appropriate *in vitro* models that recapitulate the *in vivo* environment may potentially facilitate future clinical administration. So far, the only successful clinical trial of low dose NO for biofilm inhibition was conducted in CF patients targeting *P. aeruginosa* aggregates (Howlin et al. 2017), and whether it can disaggregate other biofilms in other types of patients awaits to be evaluated.

**Table 1:** The efficacy of NO on the biofilms formed by leading pathogens in different diseases with chronic lung infections

Species	NO resources	Biofilm reduction?	Biofilm culture method
*P. aeruginosa*	• 25 nM to 2.5 mM SNP (Barraud et al. 2006); • 20 μM MAHMA NONOate (Barnes et al. 2013); • 250 μM Spermine NONOate (Cai and Webb 2020); • 256 μM DEA-C3D (Soren et al. 2019); • NO-loaded alginate beads (∼0.1–0.3 μmol/mg) (Ahonen et al. 2019a); • NO-releasing cyclodextrins (Rouillard et al. 2020b); • ∼15 mM acidified sodium nitrite (Major et al. 2010); • NO-emitting nanoparticles (Hetrick et al. 2009); • 10 ppm NO inhalation (Howlin et al. 2017)	Yes	Plate (M9 minimal medium, MH medium); Artificial sputum medium; CF patients
*S. aureus*	• Dressings producing >200ppmv gaseous NO (Sulemankhil et al. 2012); • 60 mg/ml isosorbide mononitrate (ISMN) (Hasan et al. 2018) or ISMN encapsulated in liposome (Jardeleza et al. 2014); • 125–1000 μM DETA NONOate (Jardeleza et al. 2011); • NO-loaded alginate beads (∼0.1–0.3 μmol/mg) (Ahonen et al. 2019a); • >15 mM acidified sodium nitrite (Major et al. 2010); • NO-emitting nanoparticles (Hetrick et al. 2009)	Yes	Plate (tryptic soy broth, MH medium, cerebrospinal fluid (CSF) broth); artificial sputum medium
NTHi	50 μM cephalosporin-3′-diazeniumdiolate (PYRRO-C3D/DEACP) (Walker et al. 2017)	No, but potentiates the efficacy of antibiotic	Plate (BHI medium); coculture with primary respiratory ciliated epithelial cells
*B. cepacia*	• NO-loaded alginate beads (Ahonen et al. 2019a); • >15 mM acidified sodium nitrite (Major et al. 2010)	Yes	Plate (MH medium); Artificial sputum medium
*S. pneumoniae*	100 μM and 1 mM SNP (Allan et al. 2016)	Yes, 1 mM SNP reduced the viability of cells, and 100 μM potentiates the efficacy of antibiotic	Plate (1:5 diluted BHI broth)
*M. catarrhalis*	1 mM nitrite (Mocca et al. 2015)	Yes, NO radical derived from nitrite reduction kills cells in biofilms	Coculture with human bronchial epithelial cell

### NO potentiates the efficacy of conventional antimicrobials

As the biofilm growth mode offers bacteria significant recalcitrance to antimicrobials, reversing the embedded cells back to planktonic state by NO can increase their susceptibility towards treatments. Previous studies showed that *P. aeruginosa* biofilms formed on catheters can tolerate >100 μl/ml gentamicin, colistin, chloramphenicol, ciprofloxacin, and tetracycline. However, when combined with NO, >3-log reduction of viable biofilms cells was observed when antibiotics were administrated only at 10 μl/ml (Ren et al. 2016). Nanoparticles that simultaneously release both gentamicin and NO can decrease the viability of *P. aeruginosa* biofilms by 90% at concentrations of 10–50 μM, which was much more efficient than stand-alone applications of either agent (Nguyen et al. 2016). Low dose NO significantly enhanced the efficacy of tobramycin, colistin and tobramycin+ceftazidime against *in vitro* biofilms formed by CF-PA isolates or *ex vivo* aggregates in CF sputa, suggesting a great clinical potential of this combinational therapy (Soren et al. 2019; Howlin et al. 2017). The performance of several other antimicrobials in *P. aeruginosa* biofilms, including SDS, H_2_O_2_ and human β-defensin 2, was also increased in the presence of NO (Barraud et al. 2006; Ren et al. 2016). The remaining CFU of *S. aureus* biofilms was reduced by 3-log when challenged with NO+ciprofloxacin compared to the antibiotic treatment alone (Hasan et al. 2018). Using a NTHi biofilm and PCD ciliated epithelial cells coculture model, Walker et al. reported that the susceptibility of NTHi biofilms towards azithromycin was boosted by NO, despite the same NO dosage *per se* failed to reduce the CFU of NTHi biofilms (Walker et al. 2017). Apart from reducing the tolerance in biofilms, NO can also improve the susceptibility of planktonic, multidrug-resistant pathogens towards different antimicrobials. For instance, NO-releasing chitosan oligosaccharides pretreatment resulted in a 2 to 4-log reduction in the viability of planktonic *P. aeruginosa*, MRSA and *B. cepacia*, as well as biofilms formed by multidrug-resistant *P. aeruginosa* isolates, compared to tobramycin exposure alone (Rouillard et al. 2020c). A prominent synergistic effect of NO and silver sulfadiazine was observed for many pathogens, including *P. aeruginosa* and MRSA (Privett et al. 2010). A combination of NO and superoxide resulted in a 1000-fold reduction of CFU count in planktonic *B. cepacia* compared to either component alone (Smith et al. 1999). Hence, exogenous NO presents a promising adjunctive therapeutic strategy in chronic lung infection management, either by enhancing the bactericidal effect of antimicrobials against individual cells entering the airway before biofilm formation, or reducing the tolerance of established biofilms/aggregates.

## NO delivery *in vivo*

**Fig. 3:**
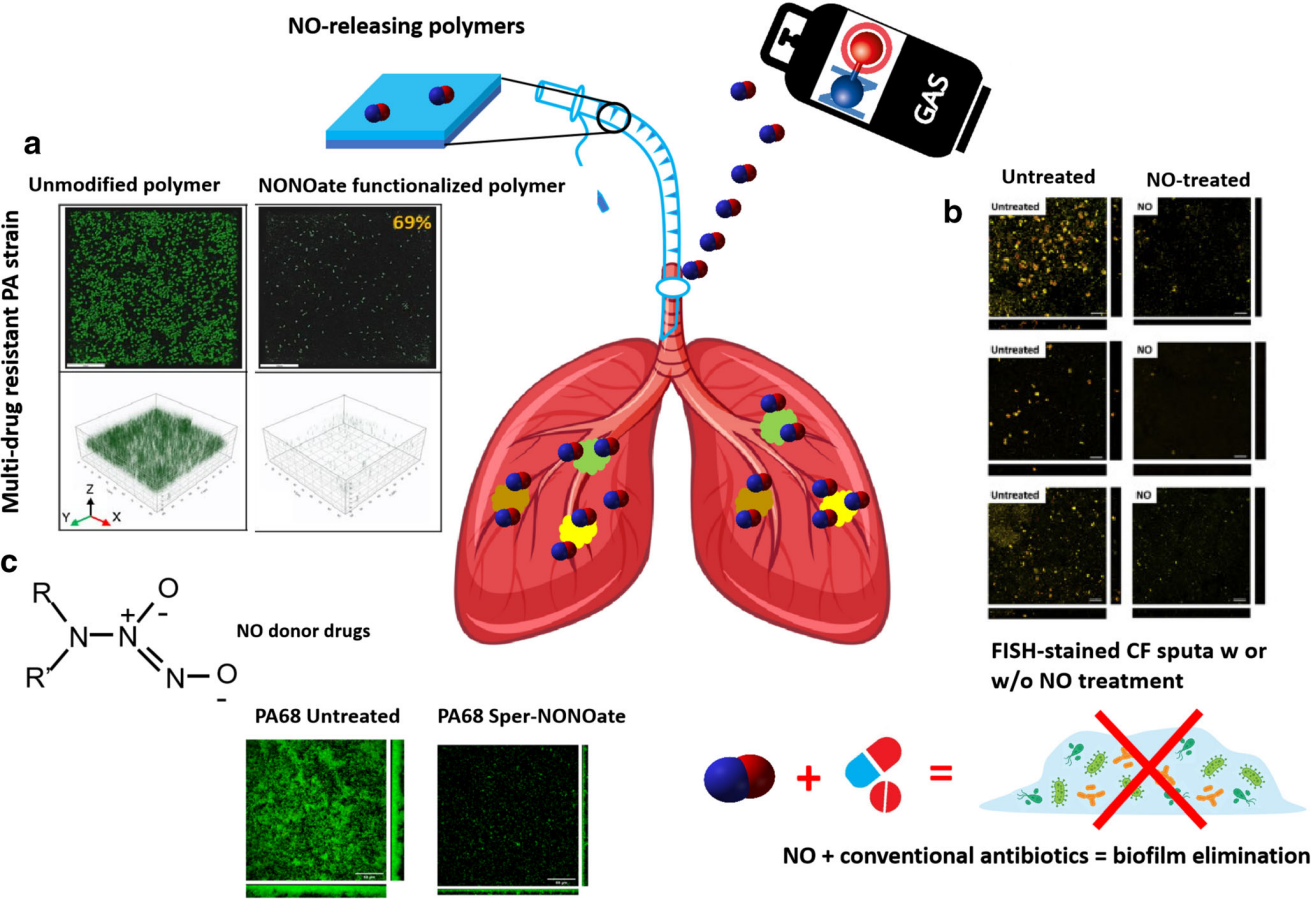
Potential methods for *in vivo* NO delivery and biofilm inhibition. **a** Significantly reduced biofilm formation of a multidrug-resistant *P. aeruginosa* strain on NONOate-functionalised polymers. Scale bar=20 μm (Sadrearhami et al. 2019). **b** NO-induced *P. aeruginosa* biofilm dispersal in CF sputum samples. *P. aeruginosa* was stained using fluorescence in situ hybridization (FISH) with both a Cy3-labeled *P. aeruginosa*-specific 16S rRNA probe (green) and a Cy5-labeled eubacterial 16S probe (red). Scale bars=25 μm (Howlin et al. 2017). **c** Biofilms formed by a CF-PA isolate can be efficiently dispersed by 250 μM Spermine NONOate (Cai and Webb 2020). The icons of lung, gas cylinder, antibiotic drugs, and bacteria in biofilms were obtained from the vectors available from Vecteezy.com. Permissions to reuse images were obtained from journals and/or Copyright Clearance Centre.

Different devices for NO delivery may significantly influence the clinical outcome. The administration of NO in patients is not a new concept—the earliest medical application of NO dates to 1879, where nitroglycerin was found able to relieve angina (Murrell 1879). The increasing discoveries of the biological activities of NO triggered a growing interest in chemist/pharmacist for the searching of new NO releasing agents and novel NO delivery strategies, as some traditional donors were proven to be toxic or carcinogenic. Furthermore, due to the short half-life and cytotoxicity effect of high-dose NO, inappropriate administration time, uncontrolled NO release or over dosage may lead to deleterious side effects and injuries (Asmawi et al. 1999; Weinberger et al. 1999; Murakami et al. 1997; Korde Choudhari et al. 2013). As such, the development of novel NO-releasing strategies for better delivery and less side effects, still remains a challenge. In the context of chronic lung infection, the involvement of pathogens adds another layer of complication—what is the optimal dosage that can remove the biofilms without triggering undue side effects? Which NO donor possesses an appropriate degradation half-life that allows released NO to be available in the vicinity of biofilms *in vivo*? Apparently, there is still a long way to go, given the fact that listed studies in Table 1 were mostly conducted *in vitro*. In this section, we summarize some traditionally and recently developed NO delivery methods (Fig. 3), hoping to stimulate more advanced research for NO-mediated bacterial inhibition in chronic lung infection.

### Inhalation of gaseous NO

Inhaled nitric oxide gas was approved by FDA in 1999 for the treatment of hypoxic infants (Nelin and Potenziano 2019). Some commercial devices such as INOMAX® and Genosyl have been developed and approved by FDA for NO inhalation, which have facilitated the administration. Previous studies showed that inhalation of NO helped clear lung infections in rats infected by *P. aeruginosa* and reduced the bacterial load by 1.7–2-log (Webert et al. 2000; Jean et al. 2002). In *K. pneumoniae* infected mice, inhaling NO at lower oxygen concentration also resulted in a 5 to 10-fold reduction of CFU in BALF and lung tissues (Sun et al. 2006). Similar bactericidal effects were also observed in CF patients, where case studies showed that intermittent administrations of 160 ppm NO significantly reduced CFU of *P. aeruginosa*, *S. aureus*, and *B. multivorans* in CF patients that are resistant to multiple antibiotics (Deppisch et al. 2016; Bartley et al. 2020). As such, inhalation of NO may improve the therapy of chronic lung infections in CF patients, particularly for those suffering from MDR pathogens. Although the above reports showed that such high dosages of NO were well-tolerated in tested patients, they may bring along toxic side effects to others. For instance, after high dosages (≥80 ppm) of NO inhalation, methemoglobinemia can be observed, and the high concentrations of toxic nitrogen dioxide generated from spontaneous oxidation result in direct injury to the alveolar epithelial cells and may even cause pulmonary oedema (Davidson et al. 1998; Kido et al. 2017). In addition, the toxic reactive nitrogen intermediates such as peroxynitrite generated under conditions of high-NO flux may induce structural alterations in DNA and cell damage, interfere with the activity of lung surfactants and increase surface tension (Hallman and Bry 1996). As the metabolic fate and systemic effects of inhaled NO can be largely affected by airway chemistry and environment, the administration of high dose NO needs careful evaluation in different patients. Whilst the applications of such high dosage were mainly due to the bactericidal effect of NO, the first clinical trial with low-dose NO inhalation (5-10 ppm) combined with standard antibiotics (ceftazidime and tobramycin) also reduced the number of *P. aeruginosa* biofilm aggregates by 3.5-log in 12 CF patients (Howlin et al. 2017). These results indicated that low dose NO may be used as adjunctive therapy for CF-related biofilm infections, especially when high-dosage NO inhalation causes severe toxicological effects.

Inhalation of NO (lower than 80 ppm) has also been applied in COPD patients for reducing pulmonary vascular resistance (Ashutosh et al. 2000; Hajian et al. 2016; Yoshida et al. 1997). However, the relationship between inhaled NO and bacterial load in COPD patients have not been investigated yet. As the existence of biofilm is yet to be confirmed in these patients, no study was carried out to evaluate if such lower dose of NO in COPD patients can inhibit biofilm aggregates. Future studies on NO applications in COPD patients with pulmonary hypertension may consider including sputum culture data for determining a possible change in bacterial load. Inhalation of NO has not been a favoured device for NO augmentation in PCD patients so far. It is therefore not yet known if it is appropriate to administrate NO in PCD patients through inhalation, and if suitable can it also reduce bacterial load and inhibit biofilms. Further investigations are needed to address these questions.

### NO-releasing compounds (NO donors)

Some classic NO donors proven by FDA such as SNP, nitroglycerin (NTG), and isosorbide dinitrate (ISDN), have long been used in a variety of clinical settings in cardiovascular diseases (Hottinger et al. 2014; den Uil and Brugts 2015; Nyolczas et al. 2018). Yet, some side effects rising from the constant usage of organic nitrates and SNP were observed. For example, a long-term supply of nitroglycerin may cause nitrate tolerance, increase oxidative stress, and induce endothelial dysfunction (Daiber and Münzel 2015; Sydow et al. 2004; Schulz et al. 2002; Hink et al. 2007). This tolerance was observed in almost all organic nitrate compounds, albeit to different extent. Much less tolerance was found in the usage of SNP. However, the decomposition of SNP releases cyanide, and the toxicity as a result of cyanide accumulation concurrent with the nitroprusside administration is well documented in clinical cases, even when the dosage was lower than recommended (Davies et al. 1975; Chung et al. 2016; Udeh et al. 2015; Rindone and Sloane 1992). Such side effects triggered the particular interest in other two classes of NO donors, diazeniumdiolates (NONOates) and S-nitrothiols, due to their distinct advantages. NONOates are a series of compounds containing a X-[N(O)NO]- functional parent nuclear structural unit, where X represents different amines or polyamines. They can spontaneously decompose to generate up to 2 moles of NO per mole of donors with first order release kinetics under physiological conditions. The half-lives of NONOates at body temperature and pH largely depend on the chemical structures of amines, which exhibit a wide range that covers different demands. Moreover, after NO release, there is no other metabolites produced except for the original amines (For a more detailed review of NONOates please see Li et al. 2020). However, such spontaneous systemic release can lead to enormous off-target effects, and it is hard to precisely control the concentrations of NO at a desired location during real administrations. The unintended NO release or reactions, especially at high concentrations, may cause harmful or even cytotoxic effect in different cell types. The subsequent reactions between decomposition products could also lead to the formation of carcinogenic nitrosamine *in vivo* (Maragos et al. 1991). As such, the long-term safety of these compounds needs careful confirmation. Most NONOates are still at experimental stage despite the great promise, and there can be big gaps between laboratory and *in vivo* results (Chen and Zhang 2013).

In contrast, the chemistry of NO release from S-nitrosothiols is very complex. This class of compounds (general formula RSNO) contain a nitroso group attached by a single chemical bond to the sulphur atom of a thiol, where R denotes a cysteine-containing peptide (Patel et al. 2017). S-Nitrosoglutathione (GSNO) is endogenously produced by many tissue cells in human, whilst both *in vitro* and *in vivo* studies suggested that some other S-nitrosothiols are also unlikely to exhibit significant cytotoxicity when used at pharmacologically relevant concentrations (Miller et al. 2000). NO release can be triggered by a variety of factors such as light, heat, metals, other thiols, and different enzymes. Particularly, these compounds can directly transfer NO^+^ species to another thiol *via* transnitrosation reaction, which may lead to altered enzyme or receptor activity (Singh et al. 1996). Such flexible metabolic pathways will unlikely trigger tolerance after long-term usage (Miller and Megson 2007). On the other hand, however, the stability of RSNOs *in vivo* is difficult to predict by *in vitro* experiments. The number of thiol-containing enzymes, the presence of trace metals, as well as the levels of intracellular thiol glutathione, can be influenced by many disease states (Tullett et al. 2001). As RSNOs offer many advantages over the current FDA-approved NO donors, some studies focused on manipulating the chemical properties of R groups that can increase the stability of existing compounds (for more details please, see reviews by Richardson and Benjamin (2002) and Al-Sa'doni and Ferro (2000)).

Although NONOates and S-nitrosothiols are greatly favoured, some other types of NO donors are still attracting much attention. Some NO donors at experimental stages, investigational stage or approved by FDA that belong to 14 major classes of NO donors are listed in Table 2, along with their status in biofilm-related research. This selection is based on their potential clinical application for biofilms in chronic lung infections. Another common class of NO donors, nitroso compounds, is not included due to their carcinogenic features (Eichholzer and Gutzwiller 1998). Whilst these NO donors offer great potential in future markets, many more investigations are required for their side effects and dosage windows, especially if they are appropriate to be applied in chronic lung infections.

**Table 2:** The clinical applications and the biofilm inhibition efficacy of 14 major classes of NO donors. *A*, approved drugs, drugs that have been officially accepted for commercialization in at least one jurisdiction at a given time. *I*, investigational drugs, which are being researched for a determinate condition and have reached clinical trials. *E*, experimental drug (drug in discovery or prediscovery phase). These drugs are being actively pursued but have not entered clinical trials. They exhibit drug-like properties but have not been formally considered as a drug candidate. Information on A, I, and E stages of drugs is obtained from DrugBank. "Disperse" refers to (I) a transition from sessile biofilm cells to motile planktonic cells, where the CFU of remaining biofilms is decreased, and the planktonic cells in the culture medium/flow cell effluent are increased proportionally; (II) a reduction of biomass/biovolume in the remaining biofilms as indicated by microscope/crystal violet staining/CFU counts. "Inhibit" refers to a prevention of biofilm formation on the surfaces, measured by microscope/crystal violet staining/CFU counts

**Class**	**Representative compounds**	**FDA category**	**Efficacy against biofilms?**
Nitrates	Nitroglycerin	A, I	Yes, inhibit *S. aureus* biofilms (Abbas et al. 2019)
	Isosorbide dinitrate	A, I	unknown
	Isosorbide mononitrate	A	Yes, disperse *S. aureus* biofilms (Hasan et al. 2018; Jardeleza et al. 2014; Zhang et al. 2019b)
	Erythrityl tetranitrate	A, E, I	unknown
	Pentaerythritol tetranitrate	A	unknown
	Isosorbide	A, I	Inhibit dental biofilms (Beauté by Roquette® PO 500)
	Propatyl nitrate	E, I	unknown
	Methylpropylpropanediol dinitrate	E	unknown
	Tenitramine	E	unknown
	Trolnitrate	E	unknown
	Nitrate	E, I	Yes, inhibit *Burkholderia pseudomallei* biofilms (Mangalea et al. 2017)
Nitrite	Amyl nitrite	A	Unknown
	Sodium nitrite	A, I	Yes, kill/inhibit *P. aeruginosa, S. aureus, B. cepacia, S. epidermidis, K. pneumoniae and Enterobacter cloace* biofilms (Major et al. 2010; Zemke et al. 2014; Schlag et al. 2007; Kishikawa et al. 2013)
	isobutyl nitrite	A	Unknown
Metal-NO complexes	Nitroprusside	A	Yes, inhibit/disperse *P. aeruginosa, S. aureus, B.cepacia, E.coli, N. gonorrheae* and *S. epidermidis* biofilms (Barraud et al. 2009a, b)
Diazeniumdiolates (NONOates)	Spermine NONOate	E, animal models (Li et al. 2020), promising anti-cancer drug	Yes, inhibit/disperse *P. aeruginosa* biofilms (Cai and Webb 2020)
	MAHMA NONOate		Yes, disperse *P. aeruginosa, E.coli O157:H7* and *Salmonella enterica* biofilms (Barnes et al. 2013; Marvasi et al. 2014)
	Proli-NONOate		Yes, disperse multispecies biofilms on reverse osmosis membranes (Barnes et al. 2015)
	DEA-NONOate		Yes, disperse *Salmonella enterica* biofilms (Marvasi et al. 2014)
	DETA-NONOate		Yes, disperse *S. aureus* (Jardeleza et al. 2011) and multispecies biofilm on biofouled membranes (Oh et al. 2018)
	DPTA-NONOate		Yes, disperse *P. aeruginosa* biofilms co-cultured with epithelium cells (Zemke et al. 2020)
	PAPA-NONOate		Yes, disperse multispecies biofilm on biofouled membranes (Oh et al. 2018)
Nitrosothiol	SNAP (S-Nitroso-N-acetyl-d,1-penicillamine)	E	Yes, disperse *P. aeruginosa* (Barraud et al. 2006), *S. enterica*, pathogenic *E. coli* and *Listeria innocua* biofilms (Marvasi et al. 2016)
	SNVP (S-Nitroso-N-valeryl penicillamine)	E (isolated femoral arteries from rat)(Miller et al. 2000)	unknown
	GSNO (S-nitroso-glutathione)	Clinical trial (Liu et al. 2020)	Yes, disperse/kill/inhibit *P. aeruginosa, A. baumannii* and *S. aureus* biofilm (surface/nanoparticle) (Barraud et al. 2006; Lee et al. 2020; Paricio et al. 2019)
	SNOC (S-Nitrosocysteine)	E (animal model, potent anti-platelet agent) (Stuesse et al. 2001)	unknown
	SNAC (S-Nitroso-N-acetyl-cysteine)	E (animal model) (De Oliveira et al. 2006)	unknown
	HomocysNO (S-Nitrosohomocysteine)	E (animal model) (Jansen et al. 1992)	unknown
	RIG200	E (animal model and clinical trials) (Megson et al. 1997; Sogo et al. 2000)	unknown
Furoxan derivatives	Ipramidil	E (isolated guinea pig working heart) (Feelisch et al. 1992)	unknown
	4-methyl-3-phenyl sulfonylfuroxan	E (Ghigo et al. 1992)	unknown
	C92-4609 (4-hydroxymethyl-furoxan-3-carboxamide, CAS 1609)	E (isolated rabbit femoral artery and jugular vein) (Hecker et al. 1995)	Unknown
	C92-4678(4-phenyl-furoxan-3-carboxylic acid (pyridyl-3-yl-methyl)-amide)		unknown
	C92-4679 (3-phenyl-furoxan-4-carboxylic acid (pyridyl-3-yl-methyl)-amide)		Unknown
	C93-4759 (3-hydroxymethyl-furoxan-4-carboxamide)		unknown
	4-(phenylsulfonyl)-3-{[(2-dimethylamino)ethyl]thio}furoxan)	NA	Yes, disperse *P. aeruginosa* biofilm (Poh et al. 2017)
	3-formyl-4-phenyl-1,2,5-oxadiazole N2-oxide and 3-carbonitrile-4-phenyl-1,2,5-oxadiazole N2-oxide	E (animal model, antitumor) (Aguirre et al. 2006)	unknown
	(E)-4-(4-((2-isonicotinoylhydrazono)methyl)phenoxy)-3-(phenylsulfonyl)-1,2,5-oxadiazole 2-oxide (Novel furoxan derivative)	E (animal model against TB) (dos Santos Fernandes et al. 2016; de Souza et al. 2020)	unknown
	20 water soluble furoxan derivatives	E (isolated thoracic aortas from rat) (Sorba et al. 1997)	unknown
	benzodifuroxan and benzotrifuroxan	E (C6 cells and isolated aorta strips from rat) (Medana et al. 1999)	unknown
Sydnonimines	Molsidomine	Not approved by FDA but available in Europe (Kwon and Rosendorff 2018)	Yes, disperse *S. enterica, E. coli O157:H7*, *L. innocua* and *Pectobacterium* biofilms (Islam et al. 2020; Marvasi et al. 2014)
	Linsidomine	E (clinical trial, patients with erectile dysfunction)(Stief et al. 1992; Truss et al. 1994)	unknown
	Pirsidomine	E (animal model, pig and dog) (Martorana et al. 1994; Wainwright and Martorana 1993)	unknown
N-hydroxyguanidines	N-(4-chlorophenyl)-N′hydroxyguanidine	E	unknown
	N-butyl-N′-hydroxyguanidine	E	unknown
Hydroxyureas and derivatives	Hydroxyurea	A	Yes, **induce** *P. aeruginosa* biofilm through SOS stress response (Gotoh et al. 2010)
	(R)-(+)-N-[3-[5-[(4-fluorophenyl)methyl]-2- thienyl]-1-methyl-2-propynyl]-N-hydroxyurea (ABT-761, Atreleuton)	I (clinical trials terminated at phase III)(Brooks et al. 1995; Reid 2001)	unknown
Hydroxylamine and derivatives	N-methyl-hydroxylamine	NA	Yes, inhibit/disperse *P. aeruginosa* biofilms (Julián et al. 2015)
		NA	Yes, inhibit/disperse *P. aeruginosa, S. aureus,* and *E. coli* biofilms (Miret-Casals et al. 2018)
Oximes	cyclohexanone oxime	Hematotoxic in rats (Derelanko et al. 1985; Glover et al. 1999)	unknown
	4-ethyl-2E-(hydroxyimino)-5-nitro-3E-hexenamide (FK-409)	E (rat aortic transplant model) (Fukada et al. 2000; Zhang et al. 2000)	unknown
1,2-diazetine 1,2-dioxides (DD)		E (except for Ie, most DD derivatives reduce the spasm of isolated rat aorta and the arterial pressure in hypertensive rats) (Shvarts et al. 1994)	unknown
Oxatriazole-5-imine	GEA 3162 and GEA 3175 (mesoionic 3-aryl substituted oxatriazole-5-imine derivatives) (Kankaanranta et al. 1996; Karup et al. 1994)	E (rat neutrophils, rabbit aortic endothelial cells, isolated pig trachea, rat bronchi and bovine and human small bronchioles) (Taylor et al. 2004; Laursen et al. 2006; Elmedal et al. 2005; Hsu et al. 2006; Malo-Ranta et al. 1994)	unknown
Heterocyclic *N*-Oxides	N–O moiety can elicit nitric oxide-like functions(Mfuh and Larionov 2015). Minoxidil,kopexil, N,N-diallylmelamine etc	Minoxidil is at A and I stage	unknown

### NO donor prodrugs (NO-drug hybrid)

The side effects of the abovementioned traditional NO donors triggered a novel approach taking the most advantage of NO whilst minimizing toxicity - attaching a NO-releasing moiety to an existing drug. Different hybrid compounds were produced to offer various drug actions with synergistic effects, which may also reduce side effects of parent compounds and slow down NO release. So far, hybrid drugs with nitrate (nitric-oxide-releasing nonsteroidal antiinflammatory drugs, NO-NSAIDs), S-nitrosothiols (nitrosylated α-adrenoreceptor antagonists moxisylate ,S-NO-moxisylate; Diclofenac derivatives containing S-nitrosothiols, S-NO-diclofenac), NONOates (NONO-aspirin and NONO-indomethacin) and furoxan (furoxan-nicorandil) have been developed, showing great clinical potency (Fiorucci et al. 2001; Sáenz De Tejada et al. 1999; Bandarage et al. 2000; Velázquez et al. 2008; Mu et al. 2000). Apart from the applications in cardiovascular and inflammatory diseases, NO donor prodrugs that are linked to antimicrobials or can be specifically activated by bacterial enzymes have attracted much attention from medical microbiologists. For instance, a combination of metronidazole (and its amino analogues) with furoxan and furazan moieties yielded some compounds that exhibited higher antibacterial efficacies against *Helicobacter pylori*, even for clinical isolates that resist metronidazole (Bertinaria et al. 2003). Considering the spontaneous decomposition of NONOates, Chen et al. synthesized β-galactosylpyrrolidinyl diazeniumdiolates (β-Gal-NONOate), which showed much higher specificity and bactericidal activity towards *E. coli* engineered to express β-galactosidase (Chen et al. 2006). However, these compounds did not solve the problem of low specificity against different clinical isolates in the complex *in vivo* environment*.* Following the concept of using bacteria-specific enzymes, a prodrug linking a diazeniumdiolate moiety to a β-lactam analogue cephalosporin, Cephalosporin-3′-diazeniumdiolates (DEACP/C3D/PYRRO-C3D), was developed (Barraud et al. 2012). This compound remains highly stable in solution and only releases NO upon the activation of bacteria-specific enzyme β-lactamase. *In vitro* studies showed that DEACP can effectively inhibit *P. aeruginosa* biofilms, including those formed by clinical isolates, and increased the susceptibility of *P. aeruginosa* and NTHi biofilms against antibiotics (Soren et al. 2019; Barraud et al. 2012; Collins et al. 2017). Furthermore, the effective concentration of DEACP against biofilms exhibits no cytotoxicity when tested on murine fibroblast cells (Barraud et al. 2015), showing great potential for clinical trials. Despite the necessity of further tests in mucus and animal models, this method of drug hybridization based on broad-spectrum, bacteria-specific enzymatic activities and NO donors may significantly increase the specificity of NO delivery towards pathogens surrounded by a myriad of biological targets in chronically infected lungs.

### NO-releasing polymeric materials

Synthetic and hybrid polymers have been used for multiple medical applications. Due to their tunable physical and chemical properties by modifying synthetic precursors and procedures, they can be designed to load many different molecules matching specific requirements (Maitz 2015). Polymers are also proven to be efficient platforms for NO, with excellent NO storage stability and prolonged NO release. Furthermore, they can also be easily loaded with other antimicrobial substances to achieve synergistic effects (Rong et al. 2019; Namivandi-Zangeneh et al. 2018). Numerous NO-releasing polymers have been developed in recent years for antimicrobial and antibiofilm effects, either in the format of small-sized nanoparticles as drug vectors to improve solubility and tissue specificity, or as surface coating for in-dwelling medical devices (Rong et al. 2019). For instance, polyvinyl chloride impregnated with S-nitroso-N-acetylpenicillamine (SNAP) significantly reduced the colonisation of *E. coli* and *S. aureus*, with excellent storage stability and ease in preparation (Feit et al. 2019). A core cross-linked star polymer containing poly(oligoethylene methoxy acrylate) and encapsulated NONOates significantly prevented the formation of *P. aeruginosa* biofilms and can greatly convert established biofilms into planktonic cells at concentrations that are nontoxic to bacteria (Duong et al. 2014b). However, the cytotoxicity of these two types of polymer against human cell lines was not investigated. Metal nanoparticles coated with NO-releasing polymers were also developed, such as AuNP@P(OEGMA)-b-P(VBHA/NO) prepared by grafting NONOate functionalised poly((oligoethyleneglycol methyl ether) methacrylate)-block-poly(vinyl benzyl chloride) onto Au nanoparticles. This polymer/gold hybrid nanoparticle constantly released NO at a slow rate for 6 days, resulting in an 83% reduction in the biomass of *P. aeruginosa* biofilms (Duong et al. 2014a). However, when tested against human cell lines, these NO-loaded nanoparticles significantly decreased the viability of both cancer and noncancerous cells. Although the reason behind such cytotoxicity was not evaluated, earlier studies raised the concerns with diazeniumdiolated-based polymers, where NO-donors were found to leach from the polymers and potentially result in the formation of carcinogenic N-nitrosoamines (Annich et al. 2000; Mowery et al. 2000). This leach also suggested the issues with sustained NO release and the stability of the NO donor within this type of polymers, which could limit shelf life or ability to be sterilised. Therefore, covalent attachment of the diazeniumdiolate group to the polymer backbone was deemed as a better approach in the development of new NONOate-conjugated polymers.

Whilst the abovementioned polymers tend to release NO at a relatively low concentration aiming at inhibiting biofilm formation, polymers storing a large volume of NO may eradicate biofilms with its bactericidal function. A copolymer consisting of cross-linked branched polyethylenimine (bPEI) onto N-carboxy propionyl chitosan sodium bear many secondary amines for NO loading, and an overall release amount of NO at 2.031 μmol/mg yielded prominent antibacterial function against *E. coli* and *S. aureus* (Ji et al. 2017). Alginate and chitosan are ideal platforms for inhalation applications due to their high solubility, compatibility for nebulization and little toxicity (Lee and Mooney 2012; Ahmad et al. 2005; de Jesús Valle et al. 2008; Hall et al. 2020). Nitric oxide (NO)-releasing alginates with a NO storage of 0.1–0.3 μmol/mg were highly antibacterial against *P. aeruginosa*, *B. cepacia and S. aureus* cultured in artificial sputum medium, resulting in a 5-log reduction in biofilm viability within 24 h (Ahonen et al. 2019a). NO-releasing chitosan oligosaccharides (COS-EA/NO) significantly reduced the viability of *P. aeruginosa* and *S. aureus* planktonic cells, as well as eradicated *P. aeruginosa* biofilms (Hall et al. 2020). Both NO-loaded alginate and chitosan exhibited negligible toxicity to human epithelial lung cells A549, and the latter showed much better performance than NO gas in buffered solutions, mucus, and artificial sputum (Hall et al. 2020; Rouillard et al. 2020a; Ahonen et al. 2018). Although direct inhalation of NO gas has been widely used as aforementioned, the high reactivity and short lifetime of NO in biological media, as well as the necessity in phase transition for diffusion into solutions, can hinder its total efficacy *in vivo*. In contrast, the similarly inhalable NO-releasing alginate and chitosan release their NO payload directly into the solution and only when in contact, allowing for more targeted and effective treatment. Moreover, as chitosan scaffold is positively charged, it promotes specific target against negatively charged bacteria and biofilm (Hall et al. 2020). Apart from the bactericidal effect of NO, COS-EA/NO was found to decrease the viscoelastic moduli and weaken the mechanical properties of biofilms (Reighard et al. 2015; Rouillard et al. 2020a), whilst both NO-releasing chitosan and alginate were shown to disrupt the mucus and reduce the viscosity and elasticity of sputum by degrading of the mucin and DNA networks (Reighard et al. 2017; Ahonen et al. 2019b). As the thick mucus layers in CF, PCD and COPD greatly contribute to the chronic infection and restrict the diffusion of NO (Hall et al. 2019), and the high viscoelasticity of biofilms may impede pathogen clearance from the lungs and impair antibiotic action (Gloag et al. 2018; Rozenbaum et al. 2019) , the application of NO-releasing alginate and chitosan may significantly benefit the treatment of pathogens by both breaking the shield and killing the cells. Despite the need for more investigations, these polymer-based NO donors showed superior efficacies to gaseous NO in biological relevant models and hold great clinical promises.

Polymer NO donors can also be incorporated into indwelling medical devices, such as endotracheal tubes in mechanical ventilation, to prevent biofilm formation in the respiratory tract. Fluorinated derivatives of NO donor SNAP, C_2_F_5_-SNAP, and DiCF_3_Bn-SNAP, were doped into polyvinylidene fluoride (PVDF) tubes. These tubes showed significant antimicrobial and antibiofilm activities against *S. aureus* and *P. aeruginosa* (Zhou et al. 2019; Zhou et al. 2018), suggesting a great potential in the development of antibacterial biomedical devices applied in respiratory diseases.

## Concluding remarks and future perspectives

Chronic bacterial infection is the leading cause of morbidity and mortality in CF, COPD and PCD patients, which affect the life quality of millions of people around the world. Bacteria that successfully settle down in the respiratory tracts go through a series of pathoadaptation, including the formation of biofilm aggregates and genetic mutations, granting them high tolerance to many antibiotics (Winstanley et al. 2016). As a result, once chronic infections establish, they are almost untreatable. The insufficient antibiotic suppression may in return induce biofilm formation and the development of resistant genes (Hoffman et al. 2005; Ahmed et al. 2018). Hence, novel antibiofilm therapeutic strategies are urgently needed for the treatment of these patients and minimizing the transmission of superbugs in nosocomial infections.

In the airway, nitric oxide plays a vital role as proinflammatory and immunomodulatory mediator in pathophysiological conditions (Ricciardolo 2003). At high concentrations, it can exhibit bactericidal effect against invading pathogens; at low concentrations, it can revert the established biofilms back to the planktonic state, thus increasing their susceptibility towards antibiotics. Therefore, NO has been regarded as a promising adjunctive therapeutic strategy in chronic lung infections. The functions of NO *in vivo* heavily rely on the concentrations generated under specific circumstances, the location, and the timing of synthesis. As such, a thorough understanding of NO metabolism in pulmonary disease patients helps to elucidate how the dysfunction of this pathway contributes to chronic infections, and a clear picture of the pathogenesis may significantly facilitate the decision of appropriate NO applications. So far, most of the NO pathway studies in chronic lung infections focused on CF, and supplementation of NO has been shown to improve lung function and help reducing bacterial load. However, the biosynthesis of NO in PCD and COPD patients attracted much less attention, and the lack of knowledge makes it difficult to evaluate the benefit or necessity of different NO augmentation methods. Whilst NO supplementation has been conducted in PCD and COPD, and it was suspected that NO augmentation will improve treatment of bacterial infections in PCD and COPD, no clinical data is available to demonstrate a significant reduction in bacterial load. On the other hand, solid clinical observations of biofilms were only reported in CF, despite the long-term implications in PCD and COPD patients. The absence of direct observation of biofilms in the lungs or sputa samples from PCD and COPD patients further hinders the development of better strategy for NO-based treatments, when various concentrations of NO perform differently in the inhibition of biofilms *in vivo*. Future research may focus on (1) NO production and dysfunction in PCD and COPD patients in order to determine whether and how exogenous NO application would benefit these patients; and (2) clarifying the predominant bacterial lifestyle (planktonic or biofilm) and the primary location of pathogens in the respiratory tract (on epithelium or mucus) in PCD and COPD patients so that we can design better NO treatment strategy targeting different formats of bacterial growth without triggering toxic effects on the delicate tissues. The abundant literature of NO pathway and pathogenesis, as well as different detection methods of biofilms and bacterial load in CF, will shed light on similar studies in PCD and COPD patients.

Given that externally applied NO would significantly reduce bacteria in these patients, either based on its antibacterial effect at high concentrations, or antibiofilm function at low scales, the optimised design of NO delivery is crucial for successful applications. Numerous novel synthetic NO donors or polymer NO hybrid drugs have been developed in recent years, and they are primarily tested *in vitro*. Therefore, the selection of *in vitro* model greatly affects the precision of conclusion in drug development. Most chemists use type strains and routine culture medium to test their compounds, which do not take into considerations of the plasticity of clinical strains and the complicated *in vivo* environment. As it is now confirmed that most *P. aeruginosa* aggregates are embedded in the thick mucus layer in CF patients, some recent studies started to test novel compounds in artificial CF sputum medium and yielded some exciting results for bacteria clearance (Ahonen et al. 2019a; Rouillard et al. 2020b). We believe this experiment model may better recapitulate the performance of these drugs *in vivo*, as *P. aeruginosa* aggregates grown in artificial sputum mimic those isolated from patients to a large extent both physiologically and transcriptionally (Turner et al. 2015; Fung et al. 2010). As such, these NO donors that passed the tests in mucus show great potentials in future clinical trials. The only application of NO donor on biofilms in PCD was conducted in NTHi/epithelium coculture (Walker et al. 2017). Whether this model is suitable to reflect the efficiency of NO drug remains unclear due to the lack of evidence of biofilm existence *in vivo*. Again, a clear picture of how bacteria are settled in PCD and COPD is crucial for optimised NO donor design, and future NO donor research from the chemistry side should carefully consider the selection of an appropriate *in vitro* system based on clinical and biological findings. Nevertheless, with so much exciting progress in the discovery of novel NO donors, especially those that specifically release NO with bacterial triggers and from small-sized nanoparticles, future NO applications in chronic lung infection diseases may possess a significantly higher precision and efficiency with much fewer side effects.
